# Hierarchical composite endpoints in clinical trials for multimorbid older adults

**DOI:** 10.1016/j.eclinm.2025.103474

**Published:** 2025-09-02

**Authors:** Priya Vart

**Affiliations:** Department of Clinical Pharmacy and Pharmacology, University Medical Center Groningen, University of Groningen, Groningen, the Netherlands

**Keywords:** Older adults, Multimorbidity, Hierarchical composite endpoints, Patient-centered outcomes, Clinical trial design

## Abstract

Hierarchical composite endpoints (HCEs) has the potential to make clinical trials for multimorbid older adults more relevant. Unlike conventional time-to-first-event analyses—which can give more weight to outcomes of lesser relevance—HCEs rank outcomes by importance to patients and clinicians. This allows priorities such as avoiding hospital stays, maintaining quality of life, and preserving independence to shape trial results, while still accounting for conventional outcomes like mortality. Because they use information from more patients, HCEs can also increase statistical power, which is particularly useful in trials where recruiting older adults is difficult and evidence to guide treatment is limited. However, deciding the order of outcomes requires balancing patient and clinical priorities, and ensuring consistency and regulatory acceptance.

## Introduction

Multimorbidity is most prevalent in older adults. Management of multimorbidity in older adults is often complicated by competing health priorities, polypharmacy and limited life expectancy, which necessitate greater treatment personalization. Yet, paradoxically, multimorbid older adults are often underrepresented in major clinical trials which tends to include relatively younger patients with fewer comorbidities. Importantly, large outcome trials typically focus on composite endpoints driven by the time to the first event. For instance, in trials among patients with type 2 diabetes mellitus (T2DM) and chronic kidney disease (CKD), composite endpoints is driven by first event such as a 40% decline in estimated glomerular filtration rate, hospitalization, or mortality.^1^ However, this approach can overemphasize less meaningful outcomes for older patients, for whom preventing hospitalizations or dialysis may be far more important than modest changes in kidney function. Moreover, patient-reported and functional outcomes—key indicators of quality of life and independence for older adults—are often secondary or insufficiently powered in these studies. As a consequence, there has been a growing call to adapt clinical trial designs to enhance their relevance for older adults disproportionately affected by multimorbidity.

## Hierarchical composite endpoints

**Fig. 1:**
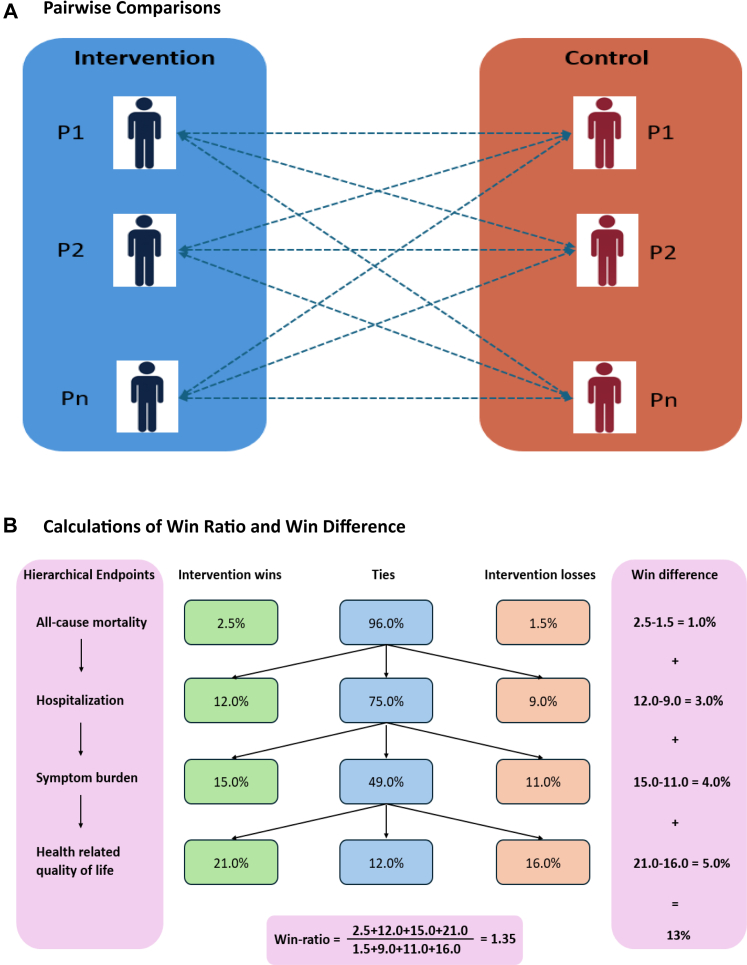
**Illustrative example of pairwise comparisons (A) and calculations of win ratio and win difference (B) in a hierarchical endpoint analysis**.

In recent years, hierarchical composite endpoints (HCEs) have gained interest in clinical trials as a method to better evaluate treatment effects in complex patient populations (Table 1).^2^ Hierarchical composite endpoints combine multiple individual outcomes into a single endpoint, with each outcome ordered by clinical priority.^3^ Starting with the highest-priority outcome, each patient in the intervention arm is compared to every patient in the control arm (Fig. 1). A “win” is determined for a given arm when the outcome does not occur or occurs later in that arm. If the outcome does not occur in either arm, or if it occurs at the same time in both arms, a “tie” is declared, and the comparison moves to the next outcome in the hierarchical order. The size and significance of treatment benefit (or harm) is then estimated by comparing the number of “wins” to the number of “losses” (Fig. 1). HCEs help overcome the limitations of traditional time-to-first-event analyses, which often capture early events that may be less clinically relevant. For example, the hierarchy may prioritize dialysis initiation, hospitalization, and/or death over modest declines in kidney function in older adults with T2DM and CKD. Similarly, hospitalization for heart failure can be prioritized over small changes in cardiac remodeling parameters in older adults with heart failure and comorbidities, allowing more clinically meaningful outcomes in these populations to have greater influence on the overall treatment effect.

**Table 1:** Examples of studies employing hierarchical composite endpoints across therapeutic areas.

	Study	Therapeutic area	Population	Intervention	Hierarchical Endpoint	Finding
1.	FINEARTS-HF^a^ (n = 6001)	Cardiology-Heart Failure	Patients with HF and mildly reduced or preserved ejection fraction Mean age 72 years Diabetes 40.6% Hypertension 88.7%	Finerenone	1) Cardiovascular mortality 2) Number of Heart failure hospitalizations 3) Number of urgent heart failure visits	Win ratio 1.17 (95% CI: 1.04, 1.32) in favor of Finerenone
2.	RESCUE BT2^b^ (n = 1177)	Cardiology-Stroke	Patients with disabling ischemic stroke without occlusion of large or medium-sized vessels Median age 68 years Hypertension 64.2% Diabetes 27.9%	Intravenous tirofiban	Median EQ-5D-5L score at 90 days	Win ratio 1.40 (95% CI: 1.23–1.62) in favor of Intravenous tirofiban
3	REPEAT^c^ (n = 3774)	Critical care	Patients undergoing general anesthesia for surgery Mean age 57 years Obesity 56.7% Cancer −41.6%	High positive end-expiratory pressure	1) All-cause hospital mortality; 2) Hospital length of stay; 3) Need for postoperative mechanical ventilation; 4) Severe pulmonary complications; 5) Mild pulmonary complications	Win ratio 1.00 (95% CI: 0.92, 1.09)
4	DARE-19^d^ (n = 1250)	Infectious disease-COVID-19	Patients with cardiometabolic risk factors and hospitalized with COVID-19 Mean age 61.4 years, Type 2 diabetes 50.9% Hypertension 84.8% With two or more cardiovascular risk factors 48.9%	Dapagliflozin	1) All-cause mortality, 2) Organ dysfunction during the index hospitalization, 3) Supplemental oxygen requirement for patients hospitalized at day 30 without organ dysfunction, 4) Hospital discharge before day 30	Win ratio 1.09, 95% CI: 0.97–1.22
5	REPLACE COVID^e^ (n = 152)	Infectious disease- COVID-19	Patients hospitalized with COVID-19 and receiving a renin–angiotensin system inhibitor before admission Mean age 62 years Diabetes 52% Hypertension 100% Dyspnoea 87%	Discontinuation of their renin–angiotensin system inhibitor	1) Days to mortality during the hospitalization 2) Days on invasive mechanical ventilation or extracorporeal membrane oxygenation 3) Days on renal replacement therapy or inotropic or vasopressor therapy 4) Area under the curve of a modified SOFA score	Win difference (rank score): 8 (95% CI: −13 to 29)

Abbreviations: FINEARTS-HF, Finerenone in Adults With Heart Failure and Left Ventricular Ejection Fraction, RESCUE-BT2, Efficacy and Safety of Tirofiban Compared with Aspirin in the Treatment of Acute Ischemic Stroke, REPEAT = Renin–angiotensin system inhibitor disContinuation and reinitiation in Patients with Acute kidney injury: a rEtrospective cohorT study, DARE-19, Dapagliflozin in Respiratory Failure in Patients With COVID-19, REPLACE COVID, Randomized Elimination and Prolongation of Angiotensin-Converting Enzyme Inhibitors and Angiotensin Receptor Blockers in Coronavirus Disease 2019, SOFA, Sequential Organ Failure Assessment.

a PMID: 40531488.

b PMID: 37256974.

c PMID: 39698861.

d PMID: 34302745.

e PMID: 33422263.

## Potential and challenges of using HCEs in multimorbid older adults

For multimorbid older adults, quality of life and functional status often hold as much significance as traditional clinical endpoints.^4^^,^^5^ Ability of HCEs to include patient-centered outcomes, such as quality of life measures and functional independence, alongside more conventional endpoints (e.g., mortality) allow studies to be powered also for more patient-centered outcomes. Importantly, HCEs can naturally incorporate patient reported outcomes—which are often continuous or ordinal—into composite endpoints alongside binary outcomes like mortality. Unlike conventional time-to-event analyses, HCEs use pairwise patient comparisons to provide greater nuance without reducing these outcomes to simple yes/no categories based on arbitrary thresholds (e.g., a decline of ≥10-point in EQ-5D VAS score), thereby preserving important information about the extent and timing of change. As a result, HCEs offers the opportunity to generate trial results that are more patient-centered and better aligned with what matters most to older, multimorbid adults. To optimally avail of this opportunity, careful selection of endpoints and thorough examination of each individual component remain essential to identify which outcomes drive the overall result and to understand the likely clinical implications of the findings. Less clinically relevant outcomes placed lower in a hierarchy can still dilute the apparent treatment effect on those that are most meaningful. For instance, if a treatment substantially improves symptom burden, its overall benefit may appear less pronounced if the composite also includes outcomes—such as modest changes in HbA1c—that may not always align in direction with the treatment's effect on more relevant outcomes and are likely of limited clinical significance to multimorbid older adults.

As newer therapies lead to declining event rates in patients with multimorbidity, future trials evaluating novel treatments will need to be larger and with a longer follow-up.^6^ Because in HCE approach more patients contribute to study endpoints, it substantially improves statistical power, and studies may be conducted with fewer patients compared to a conventional trial with time to the first event of the composite outcomes.^7^ For instance, 1000 patients in the Canagliflozin and Renal Events in Diabetes with Established Nephropathy Clinical Evaluation (i.e., CREDENCE) trial would have provided power of about only 50% in conventional approach with time to the first event of the composite outcome while the same number of patients would have provided power of over 90% when investigating HCE as a composite end point.^8^ This advantage is particularly relevant in multimorbid older adults for at least two reasons. First, the heterogeneity within the older population means that larger sample sizes would typically be required to detect treatment effects using conventional methods, compared to younger, more homogeneous populations. Second, recruiting older patients into clinical trials is often challenging due to factors like frailty and cognitive impairment and need for even larger trails in future can potentially further limit evidence for older patients, who have been historically underrepresented. This could be of particular relevance to trials in areas such as deprescribing and disease prevention in older adults (Box 1). Therefore, the HCE approach can be a more efficient and feasible way to evaluate treatments in older, complex patient groups. However, depending on the study population (e.g., low/high baseline risk of hard clinical endpoints) and trial design (e.g., length of follow-up, choice of outcomes in hierarchy), the analysis of a smaller trial may place less weight on hard clinical endpoints (such as mortality) and rely more heavily on other outcomes such as functional measures or patient reported outcomes. This may not necessarily be a disadvantage in trials of multimorbid older adults, since these outcomes are often high priority outcomes but can be of concern in open-label trials, where subjective outcomes could introduce bias, warranting careful evaluation of the study results.

Box 1 **Examples of studies that may employ proposed hierarchical composite endpoints in clinical trials for multimorbid older adults**

**Table:** 

Study population	Intervention	Possible hierarchical endpoint
Hypertensive adults ≥75 years old with systolic blood pressure <150 mmHg, polypharmacy (including multiple antihypertensive medications) and frailty	Deprescribing antihypertensives	1) All-cause mortality, 2) Serious adverse drug events requiring hospitalization, 3) Symptom burden 4) Functional capacity 5) Health-related quality of life
Community-dwelling adults ≥75 years old with diabetes, presence of at least one more cardiovascular risk factor and moderate-to-severe frailty	Sodium-glucose cotransporter 2 inhibitor	1) All-cause mortality, 2) Institutionalization 3) Hospitalization 4) Activities of daily living 5) Health-related quality of life
Adults ≥75 years hospitalized for non-surgical acute conditions (e.g., pneumonia, heart failure etc.) with ≥2 comorbidities and high risk of delirium	Proactive delirium prevention program	1) In-hospital or 90-day mortality 2) Readmission within 90 days 3) Functional capacity 4) Health-related quality of life

Competing risks are a frequent challenge in trials among multimorbid older adults, where death can preclude observation of other clinically relevant outcomes such as hospitalization, functional decline, or changes in quality of life.^9^ When all components are time-to-event measures and death is explicitly included in a conventional composite endpoint, competing risk concerns are largely addressed. However, many trials analyze continuous or ordinal outcomes (e.g., change in eGFR, quality-of-life scores) over a shorter follow-up period to demonstrate ‘proof of concept’ and/or for feasibility reasons. In these cases, conventional mean-difference analyses typically rely on data from survivors only or assign arbitrary worst scores to deceased patients, which can bias results. HCEs offer a principled alternative by allowing death to be explicitly included in the hierarchy: pairwise comparisons are resolved on death first when relevant, while continuous outcomes are compared among surviving patients using prespecified clinically meaningful thresholds. This approach preserves the power advantage of continuous endpoints, avoids survivor-only bias, and ensures that deaths are properly accounted for in the overall treatment effect estimate.

While HCEs provide flexibility, defining an appropriate hierarchy can be complex, particularly for multimorbid older adults.^10^ The order of outcomes must balance clinical significance with patient-centered goals, which may vary widely among these patients. For instance, some patients might prioritize survival while others may prefer independence and quality of life over longevity, especially if living with severe medical complications. Establishing an optimal hierarchy would require input from clinicians, patients, and caregivers, as well as a clear understanding of disease progression. A potential solution could be to construct a patient-specific hierarchy of clinical outcomes, informed by individual preferences. Data on these preferences can be collected during study participation. Based on the ranked importance of outcomes (e.g., first, second, or nth priority), weights can be assigned and subsequently incorporated into the calculation of the ‘win’ in hierarchical composite endpoints. Nonetheless, standardization and validation of outcome hierarchies are essential to enhance the acceptability of HCEs in clinical trials. To support this, regulatory agencies could establish formal validation frameworks for the components of HCEs. In parallel, trial sponsors should engage early with regulators, provide justification for the chosen hierarchy based on clinical relevance, and conduct pre-specified sensitivity analyses to assess the robustness of trial findings.

Interpretation of win statistics, such as the win ratio used to report efficacy estimates in HCE trials, is less intuitive than that of conventional effect measures like risk ratios or hazard ratios. For example, a win ratio of 1.20 means that, among all non-tied patient pairs, the probability that the treatment “wins” is 20% higher than that of the control. However, this does not mean that 20% of events are prevented, as the win ratio is not equivalent to a risk ratio. In one clinical trial of patients hospitalized for acute heart failure, the observed win ratio was 1.36 in favor of the intervention, even though the intervention had no effect on one component of the composite outcome (i.e., the quality of life score), and for the other components, the reduction in risk was less than 36%. This illustrates how the win ratio reflects the ordering and frequency of events, rather than a proportional reduction in event rates. But similar to the risk ratio, the win ratio can be high even if only a few patients respond to the intervention. To improve interpretability, additional win statistics—such as the win difference, which sums the differences in percentage wins and losses across all hierarchical components—can be reported alongside the win ratio (Fig. 1). This offers a relatively more intuitive interpretation: out of 100 randomly selected intervention–control patient pairs, how many more comparisons favor the intervention than favor the control.

## Conclusion

HCEs offer significant potential for clinical trials involving multimorbid older adults with multimorbidity, addressing the limited evidence on benefit risk balance in this population. By structuring endpoints in a clinically meaningful hierarchy, HCEs can provide a holistic view of treatment effects, improving statistical power and reflecting patient centered priorities. However, their design and interpretation come with challenges, including the complexity of defining relevant hierarchies, and interpreting composite results. Standardizing and validating HCEs in multimorbid older adults would be essential to maximize the potential of HCEs in this population.

## Contributors

P.V. reviewed the literature and drafted the manuscript.

## Declaration of interests

None declared.
